# A whole-genome screen identifies *Salmonella enterica* serovar Typhi genes involved in fluoroquinolone susceptibility

**DOI:** 10.1093/jac/dkaa204

**Published:** 2020-06-08

**Authors:** A Keith Turner, Sabine E Eckert, Daniel J Turner, Muhammud Yasir, Mark A Webber, Ian G Charles, Julian Parkhill, John Wain

**Affiliations:** d1 Wellcome Sanger Institute, Wellcome Genome Campus, Hinxton, Cambridgeshire CB10 1SA, UK; d2 Quadram Institute, Norwich Research Park, Colney, Norwich NR4 7UA, UK; d3 University of East Anglia, Norwich Research Park, Norwich NR4 7TJ, UK; Oxford Nanopore Technologies Ltd, Gosling Building, Edmund Halley Road, Oxford Science Park OX4 4DQ, UK; Department of Veterinary Medicine, University of Cambridge, Madingley Road, Cambridge CB3 OES, UK

## Abstract

**Objectives:**

A whole-genome screen at sub-gene resolution was performed to identify candidate loci that contribute to enhanced or diminished ciprofloxacin susceptibility in *Salmonella enterica* serovar Typhi.

**Methods:**

A pool of over 1 million transposon insertion mutants of an *S.* Typhi Ty2 derivative were grown in a sub-MIC concentration of ciprofloxacin, or without ciprofloxacin. Transposon-directed insertion site sequencing (TraDIS) identified relative differences between the mutants that grew following the ciprofloxacin treatment compared with the untreated mutant pool, thereby indicating which mutations contribute to gain or loss of ciprofloxacin susceptibility.

**Results:**

Approximately 88% of the *S.* Typhi strain’s 4895 annotated genes were assayed, and at least 116 were identified as contributing to gain or loss of ciprofloxacin susceptibility. Many of the identified genes are known to influence susceptibility to ciprofloxacin, thereby providing method validation. Genes were identified that were not known previously to be involved in susceptibility, and some of these had no previously known phenotype. Susceptibility to ciprofloxacin was enhanced by insertion mutations in genes coding for efflux, other surface-associated functions, DNA repair and expression regulation, including *phoP*, *barA* and *marA*. Insertion mutations that diminished susceptibility were predominantly in genes coding for surface polysaccharide biosynthesis and regulatory genes, including *slyA*, *emrR*, *envZ* and *cpxR*.

**Conclusions:**

A genomics approach has identified novel contributors to gain or loss of ciprofloxacin susceptibility in *S.* Typhi, expanding our understanding of the impact of fluoroquinolones on bacteria and of mechanisms that may contribute to resistance. The data also demonstrate the power of the TraDIS technology for antibacterial research.

## Introduction

*Salmonella enterica* subsp. *enterica* serovar Typhi causes tens of millions of cases of typhoid fever, resulting in over 100 000 deaths annually.^1^ These are likely to be underestimates because of the predominance of typhoid fever in low- to middle-income countries where there is often a paucity of diagnostic facilities^2^ capable of differentiating typhoid fever from other, clinically similar, febrile diseases.^3^ Since the introduction of chloramphenicol for the treatment of typhoid, the spread of antibiotic-resistant *S*. Typhi strains has led to treatment failures.^4^ The emergence of MDR strains associated with the acquisition of plasmids^5^ and the emergence of the H58 haplotype^6^^,^^7^ and of XDR strains has led to typhoid fever that fails to respond to treatment with any of the antibiotics commonly used for treatment, including chloramphenicol, sulfamethoxazole/trimethoprim, ampicillin/amoxicillin, ciprofloxacin and ceftriaxone.^8^^,^^9^ Furthermore, XDR typhoid fever is now spreading in Pakistan.^10^

Ciprofloxacin (and other fluoroquinolone antibacterials) target the topoisomerase enzymes, DNA gyrase and topoisomerase IV, which are essential functions required for the maintenance of appropriate levels of DNA topology. In *Escherichia coli* and *Salmonella*, resistance is multifactorial, but usually requires point mutations leading to amino acid substitutions at Ser83 of the DNA gyrase GyrA subunit.^11^ Alone, such mutations reduce susceptibility to fluoroquinolones and have resulted in prolonged treatment times with increased shedding, and outright treatment failures.^6^^,^^12–15^ However, high-level resistance requires further factors, such as additional mutations within the topoisomerase genes, or is associated with those conferring reduced cell permeability and increased antibiotic efflux, or acquisition of a quinolone resistance determinant, such as *qnr*.^8^^,^^11^^,^^16–18^

It is likely that other mechanisms exist that contribute to fluoroquinolone resistance in *S*. Typhi, which may be relevant to other bacteria, and the application of transposon insertion sequencing technologies, such as transposon-directed insertion site sequencing (TraDIS),^19–22^ provides a way to identify these other mechanisms. This involves growing a very large collection of bacterial transposon insertion mutants as a pool under a growth condition of interest. Within the population of mutants, some will show a loss of susceptibility to the condition, and grow more poorly, whilst others will show reduced susceptibility and grow relatively faster. By determining nucleotide sequences from the transposon insertions into the adjacent DNA and comparing them with a reference whole-genome nucleotide sequence, the sequence reads can pinpoint the locations of many transposon insertions in the pool of mutants simultaneously. The number of sequence reads generated at the different sites provides a semi-quantitative measure of the numbers of mutants and, by using at least several hundred thousand transposon mutants, the whole of the non-essential bacterial genome may be assayed down to a resolution of, on average, a few base pairs. Thus, TraDIS can measure changes in the population of the transposon mutants in a condition of interest compared with a standard control.^21^

In this manuscript, we describe the use of TraDIS to assay the *S.* Typhi genome for genes involved in gain or loss of susceptibility to ciprofloxacin in a strain that already harbours a *gyrA* mutation resulting in a GyrA Ser83Phe amino acid substitution and exhibiting reduced susceptibility to fluoroquinolones.^16^ Our data identify many of these genes, including those already known and new ones, thus providing clues as to which genes may mutate and contribute to increased clinical resistance. In addition, mutations that lead to a gain of susceptibility indicate that the products of these genes are candidates for the development of antibacterials that may restore susceptibility to ciprofloxacin.

To our knowledge, this is only the second whole-genome screen to investigate the pathways to gain or loss of susceptibility to a fluoroquinolone at this level of genome resolution in bacteria.^23^

## Materials and methods

### S. Typhi transposon mutant library

The transposon mutant library used for this study has been described previously and was estimated to consist of at least 1 million mini-Tn*5* transposon insertion mutants^21^ (for more details see the Supplementary data, available at *JAC* Online). The mutant collection exists, and is used, as a single pool split between experimental growth conditions, and the majority of mutants each contain a single transposon insertion in the genome. The parent strain, WT26 pHCM1, possesses a GyrA Ser83Phe substitution conferring reduced susceptibility to fluoroquinolone antibiotics (MIC of ciprofloxacin 0.25 mg/L compared with 0.016 mg/L for the parent strain), and harbours the multiple antibiotic resistance plasmid pHCM1. This strain has attenuating deletion mutations in *aroC*, *aroD* and *htrA*, and requires supplementation of LB broth with 0.004% phenylalanine, 0.004% tryptophan, 0.001% *para*-aminobenzoic acid, 0.001% dihydrobenzoic acid and 0.004% tyrosine (‘aro mix’) to grow.^16^^,^^24–26^

### Passage of the transposon mutant library pool

The *S.* Typhi transposon mutant pool (1 × 10^9^ cfu) was grown in 100 mL of LB broth + aro mix, with or without 0.05 mg/L of ciprofloxacin (about ⅕ × MIC)^16^ in duplicate. After overnight incubation at 37°C, a second passage of each of the four cultures was prepared similarly and inoculated with 1 mL of the previous respective culture, and again incubated overnight at 37°C, to give growth amounting to a total of about 15 generations. Genomic DNA was extracted from ∼1 × 10^10^ cells from each culture using a genomic DNA extraction buffer kit and Tip-100G columns (QIAGEN).

### TraDIS sequencing and data analysis

Nucleotide sequences were generated from each extracted DNA sample using the modified protocol described previously.^21^ This uses a custom oligonucleotide sequencing primer which anneals to the nucleotides of known sequence at the transposon end to generate nucleotide sequence reads from the transposon into the adjacent target DNA for all of the transposon mutants simultaneously. Nucleotide sequence reads (Table S1) were compared with a reference genome nucleotide sequence that combined the sequences of *S.* Typhi Ty2 (accession number AE014613) and plasmid pHCM1 (accession number NC_003384), using the Bio-Tradis software suite^27^ installed on CLIMB virtual machine servers.^28^ Nucleotide sequence homology between a sequence read and the reference genome locates a transposon insertion site, and the number of sequence reads that locate with that site is a semi-quantitative measure of the copy number of mutants present in the transposon mutant pool.

The mutant site and number of reads were compared with the genome annotation to determine the number of reads that locate within each gene. Comparison of the data of the controls with the ciprofloxacin-treated duplicates using the Bio-Tradis analysis software toolkit gives the ratio of log_2_ fold change (log_2_FC), log_2_ counts per million (log_2_CPM) and *P* and *q* values for each gene. The FC refers to the difference in the number of sequence reads that locate with a gene between ciprofloxacin-treated and untreated conditions. Thus, negative values indicate that insertion mutation in that gene results in a gain in ciprofloxacin susceptibility and positive values a loss. These data were filtered for genes that had log_2_ FC values ≥1.4 or ≤−1.4 (i.e. an increase or decrease of greater than 2.6-fold). CPM refers to the number of reads per gene if the total number of nucleotide sequence reads generated for the sample had been 1 million. Genes with sequence read values of log_2_ CPM <2.75 (less than 6) were discarded from the data set. The data sets presented here have at least 13 million associated sequence reads (Table S1). Thus, any gene represented by fewer than 78 reads was excluded from the analysis. The *P* value is the statistical probability of obtaining the result if the gene is not involved in gain or loss of susceptibility to ciprofloxacin, and the *q* value is the *P* value adjusted for the false discovery rate (the proportion of false positives expected from a test). Gene data filtered as above all had *q* values of <0.00002. Genes within this data set are likely to contribute the most to gain or loss of susceptibility to 0.05 mg/L ciprofloxacin and are presented in Tables 1 and 2, but do not necessarily include all candidates. Supplementary data are available with less stringent thresholds (−1 >log_2_FC >1, *q *<* *0.001; Table S2).

**Table 1.** *S.* Typhi transposon insertion mutants displaying enhanced susceptibility to ciprofloxacin^a^

Higher function	Gene name	Function	Log_2_FC	*q* value^b^
Membrane/surface associated	*acrA*	acriflavin resistance protein A; AcrAB–TolC efflux	−9.27	3.99 × 10^−74^
	*acrB*	acriflavin resistance protein B; AcrAB–TolC efflux	−6.85	2.6 × 10^−304^
	*ybhT*	putative exported protein	−3.24	9.99 × 10^−23^
	*tolC*	outer membrane protein TolC; AcrAB–TolC efflux	−3.01	1.78 × 10^−62^
	*t3146*	putative membrane protein	−2.04	2.61 × 10^−58^
	*t2964*	putative membrane protein; conserved	−1.86	3.43 × 10^−35^
	*t3276*	putative outer membrane protein; conserved	−1.75	4.27 × 10^−12^
	*mrcA*	penicillin-binding protein 1A	−1.60	5.3 × 10^−49^
	*plsC*	1-acyl-glycerol-3-phosphate acyltransferase	−1.57	1.03 × 10^−8^
	*t3147*	putative exported protein	−1.50	3.08 × 10^−19^
	*fliJ*	flagellar FliJ protein	−1.41	5.86 × 10^−12^
	*marB*	multiple antibiotic resistance protein MarB; periplasmic	−1.41	5.15 × 10^−16^
Regulators	*phoP*	transcriptional regulatory protein PhoP	−3.00	4 × 10^−29^
	*barA*	sensor protein	−2.87	2.27 × 10^−62^
	*marA*	multiple antibiotic resistance protein MarA	−2.87	7.17 × 10^−50^
	*tyrR*	transcriptional regulatory protein TyrR	−2.86	9.74 × 10^−58^
	*sirA*	invasion response-regulator	−2.58	7.03 × 10^−7^
	*cysB*	cys regulon transcriptional activator	−2.54	3.3 × 10^−172^
	*t3449*	possible LysR-family transcriptional regulatory protein	−2.51	5.59 × 10^−16^
	*phoQ*	sensor protein PhoQ	−1.94	4.78 × 10^−34^
	*fadR*	fatty acid-fatty acyl responsive DNA-binding protein	−1.55	4.87 × 10^−11^
	*rob*	right origin-binding protein	−1.41	2.88 × 10^−23^
DNA repair/nucleoid associated	*hupA*	histone-like DNA-binding protein HU-alpha	−3.57	2.66 × 10^−74^
	*recN*	DNA repair protein	−3.19	1.7 × 10^−20^
	*xseA*	exodeoxyribonuclease large subunit	−3.04	5.7 × 10^−64^
	*sbcB*	exodeoxyribonuclease I	−2.32	1.79 × 10^−85^
	*t1056*	putative ATP-dependent helicase	−2.24	1.33 × 10^−52^
	*hupB*	DNA-binding protein HU-beta	−2.11	7.37 × 10^−19^
	*endA*	endonuclease I	−1.76	1.37 × 10^−31^
	*recG*	ATP-dependent DNA helicase	−1.69	3.17 × 10^−8^
	*uvrD*	DNA helicase II	−1.42	9.16 × 10^−17^
Cell division	*nlpI*	lipoprotein; possibly cell division	−3.41	3.07 × 10^−29^
	*ftsN*	cell division protein	−1.86	1.34 × 10^−5^
	*dedD*	cell division protein	−1.66	1.15 × 10^−9^
	*ftsH*	cell division protein	−1.43	9.56 × 10^−8^
RNA/RNA processing	*trpS*	tryptophanyl-tRNA synthetase	−2.36	8.06 × 10^−17^
	*hfQ*	host factor-I protein	−1.97	3.54 × 10^−40^
	*micF*	small RNA regulator of *ompF* expression	−1.87	9.39 × 10^−7^
	*pnp*	polynucleotide phosphorylase	−1.59	1.05 × 10^−10^
Others	*t2965*	conserved hypothetical protein	−2.73	1.08 × 10^−21^
	*t0625*	tRNA-Pro	−2.00	5.12 × 10^−11^
	*t0533*	putative aminotransferase	−1.65	2.18 × 10^−9^
	*ygdD*	conserved hypothetical protein	−1.52	2.45 × 10^−7^

a Nucleotide sequence reads are generated specifically from transposon insertion sites and therefore precisely identify the site location. Log_2_FC refers to the difference in nucleotide sequence reads that locate within a gene between a ciprofloxacin-treated and an untreated culture of a pool of at least 1 million mutants. The number of nucleotide sequence reads that locate within a gene reflects the number of insertion mutants that are present for that gene.^19–22^ The values are logarithms in base 2, so negative values indicate that the number of representative mutants is less for ciprofloxacin-treated compared with untreated, and that these mutants have an enhanced susceptibility in these growth conditions.

b *q* values indicate the statistical significance of the data and are *P* values adjusted for the false discovery rate.^27^

**Table 2.** *S.* Typhi mutants displaying diminished susceptibility to ciprofloxacin

Higher function	Gene name	Function	Log_2_FC	*q* value
Carbohydrate/polysaccharide metabolism	*pfkA*	6-phosphofructokinase	2.30	1.02 × 10^−38^
	*tviC*	Vi polysaccharide biosynthesis protein, epimerase	2.16	5.5 × 10^−198^
	*rfbE*	CDP-tyvelose-2-epimerase	2.11	8.14 × 10^−94^
	*waaI*	lipopolysaccharide 1,3-galactosyltransferase	2.01	8.1 × 10^−106^
	*rfbU*	putative glycosyl transferase	1.97	1.01 × 10^−60^
	*rfbM*	mannose-1-phosphate guanylyltransferase	1.97	8 × 10^−108^
	*rfbK*	phosphomannomutase	1.94	2.5 × 10^−140^
	*waaJ*	lipopolysaccharide 1,2-glucosyltransferase	1.85	7.27 × 10^−84^
	*galE*	UDP-glucose 4-epimerase	1.83	3.38 × 10^−24^
	*rffM*	probable UDP-N-acetyl-d-mannosaminuronic acid transferase	1.81	1.44 × 10^−51^
	*rffD*	UDP-ManNAc dehydrogenase	1.75	1.99 × 10^−51^
	*rfbI*	putative reductase RfbI	1.73	2.1 × 10^−105^
	*mtlD*	mannitol-1-phosphate dehydrogenase	1.68	2.67 × 10^−72^
	*nagA*	N-acetylglucosamine-6-phosphate deacetylase	1.68	1.82 × 10^−13^
	*waaK*	lipopolysaccharide 1,2-N-acetylglucosaminetransferase	1.67	1.41 × 10^−97^
	*waaL*	O-antigen ligase	1.61	3.88 × 10^−77^
	*wecB*	UDP-N-acetyl-d-glucosamine 2-epimerase	1.59	4.44 × 10^−84^
	*pgi*	glucose-6-phosphate isomerase	1.50	4.76 × 10^−77^
	*rfbS*	paratose synthase	1.46	3.52 × 10^−6^
Regulators	*slyA*	transcriptional regulator of haemolysin E	3.19	2.8 × 10^−111^
	*emrR*	putative transcriptional regulator	2.86	5.9 × 10^−183^
	*envZ*	two-component sensor kinase EnvZ	2.21	7.41 × 10^−12^
	*cpxR*	envelope stress two-component response regulatory protein	2.09	1.48 × 10^−57^
	*t1707*	putative TetR-family regulatory protein	1.95	8.65 × 10^−88^
	*phoU*	phosphate transport system regulatory protein	1.86	6.91 × 10^−14^
	*nadR*	conserved hypothetical transcriptional regulator	1.75	1.3 × 10^−114^
	*yijC*	possible TetR-family transcriptional regulatory protein	1.58	1.89 × 10^−33^
	*cytR*	transcriptional repressor	1.57	2.17 × 10^−72^
	*rseC*	sigma-E factor regulatory protein RseC	1.53	1.81 × 10^−26^
	*crp*	cyclic AMP receptor protein, catabolite gene activator	1.51	2.55 × 10^−7^
	*gntR*	gluconate utilization operon repressor	1.45	3.34 × 10^−33^
	*gatR*	galactitol utilization operon repressor	1.45	1.37 × 10^−7^
	*spf*	small regulatory RNA	1.45	1.81 × 10^−11^
	*mtlR*	mannitol operon repressor	1.40	2 × 10^−47^
Membrane/surface associated	*ompF*	outer membrane protein F precursor	3.91	0
	*t0641*	putative outer membrane lipoprotein	2.77	7.4 × 10^−171^
	*emrD*	multidrug resistance protein D	2.26	2.9 × 10^−185^
	*dgkA*	diacylglycerol kinase	2.04	3.24 × 10^−16^
	*ppk*	polyphosphate kinase	1.95	5.5 × 10^−169^
	*lepB*	signal peptidase I	1.78	9.39 × 10^−7^
	*trkH*	trk system potassium uptake protein	1.78	5.06 × 10^−30^
	*t3816*	putative secreted protein	1.69	3.59 × 10^−56^
	*nucE*	putative secretion protein	1.57	7.26 × 10^−7^
	*yabI*	DedA-family integral membrane protein	1.54	2.16 × 10^−25^
	*yhdA*	putative lipoprotein	1.54	3.09 × 10^−64^
	*t2427*	hypothetical major facilitator family transport protein	1.47	6.28 × 10^−48^
Redox associated	*gor*	glutathione reductase	2.95	6.5 × 10^−158^
	*trxB*	thioredoxin reductase	1.62	8.04 × 10^−24^
	*t1325*	putative NADH reducing dehydrogenase	1.48	1.8 × 10^−52^
	*gshB*	glutathione synthetase	1.42	7.99 × 10^−10^
	*t1326*	putative ferredoxin-like protein, cytoplasmic membrane	1.41	2.27 × 10^−17^
Nucleoid associated	*parC*	topoisomerase IV subunit A	1.97	5.61 × 10^−17^
	*hns*	DNA-binding protein	1.51	1.14 × 10^−84^
	*hepA*	probable ATP-dependent helicase HepA	1.50	1.72 × 10^−70^
RNA/RNA processing	*rpoC*	DNA-directed RNA polymerase, beta'-subunit	1.69	3.2 × 10^−115^
	*rne*	ribonuclease E	1.61	9.9 × 10^−22^
Murein metabolism	*aspC*	aspartate aminotransferase	1.74	1.62 × 10^−5^
	*dacA*	d-alanine carboxypeptidase	1.47	1.3 × 10^−72^
	*metL*	aspartokinase II	1.45	2.31 × 10^−57^
Others	*t3103*	conserved hypothetical protein	2.76	9.4 × 10^−161^
	*guaA*	GMP synthase	2.29	2.51 × 10^−14^
	*yabC*	conserved hypothetical protein	2.22	2.04 × 10^−13^
	*yojL*	thiamine biosynthesis protein	1.87	1.99 × 10^−68^
	*efp*	elongation factor P	1.79	2.21 × 10^−11^
	*t2640*	conserved hypothetical protein	1.68	1.68 × 10^−14^
	*gidA*	glucose inhibited division protein	1.68	1.84 × 10^−9^
	*thdF*	thiophene and furan oxidation protein	1.61	8.66 × 10^−7^
	*aphA*	class B acid phosphatase precursor	1.49	1.06 × 10^−33^
	*thiS*	thiamine biosynthesis protein	1.47	2.07 × 10^−7^
	*ybeA*	conserved hypothetical protein	1.44	1.83 × 10^−10^
	*ubiB*	flavin reductase	1.44	1.1 × 10^−37^
	*ybeB*	conserved hypothetical protein	1.42	7.29 × 10^−19^
	*yhjJ*	putative zinc-protease precursor	1.41	5.29 × 10^−71^

Refer to footnotes for Table 1. Positive values for log_2_FC indicate that the number of representative mutants for each gene is greater for ciprofloxacin-treated compared with untreated, and that these mutants therefore have diminished ciprofloxacin susceptibility in these growth conditions.

### Validation of candidate genes

Many of the candidate genes identified as contributing to gain or loss of susceptibility to ciprofloxacin are confirmed by published reports (see the Results and discussion). To confirm the TraDIS data and validate the predicted role of some of the candidate genes in *S*. Typhi gain of ciprofloxacin susceptibility, five genes were selected from the list of candidates for directed inactivation. The construction of these mutations was achieved using the suicide vector method described previously.^16^ This method involved the use of intermediate constructs and the generation of mutants in independent duplication to confirm that the observed phenotype was due to the constructed mutation and was not due to changes elsewhere in the genome.

### Relative MIC determinations

Determination of relative differences in the MICs of antibiotics between the five constructed mutants and parent strain was performed in 96-well microtitre plates using dilutions of ciprofloxacin, ofloxacin and nalidixic acid in total volumes of 200 μL of LB broth + aro mix (see above). Two-fold concentration increments were used for nalidixic acid, 1.5-fold for ciprofloxacin and increments of 1.2, 1, 0.85, 0.65 and 0.5 mg/L were used for ofloxacin. Bacteria were added to a concentration of about 10^5^ cfu/mL and incubated at 37°C for 18 h. The lowest concentration at which there was no obvious visible growth was taken as the MIC. MICs were determined for two mutants generated independently for each gene under investigation, and three separate MIC determinations were performed, with the modal value being presented.

## Results and discussion

### Application of the TraDIS method to ciprofloxacin susceptibility

Modification of high-throughput sequencing methodology allows the relative numbers of each transposon mutant in a large mutant pool to be determined.^19–22^ A pool of at least 1 million *S*. Typhi Ty2 derivative transposon mutants^21^ was grown with or without a sub-MIC concentration (0.05 mg/L; about ⅕ × MIC) of ciprofloxacin, and the differences in endpoint growth of the mutants between the two conditions were observed. The average distance between different transposon insertion sites is about 10 bp, so the whole genome has been assayed at this level of resolution (Table S1). The output data from these experiments are expressed as the number of sequence reads that have an identical nucleotide sequence to a particular location in the genome. With reference to the *S*. Typhi Ty2 whole-genome annotation, the number of reads that locate within each gene may then be deduced. Whilst most mutants incorporate a single transposon insertion, each gene will, on average, be represented by dozens of different mutants within the mutant pool, and by hundreds to upwards of several tens of thousands of nucleotide sequence reads (see the Materials and methods). Because the assay was performed using a sub-MIC concentration of ciprofloxacin that allowed many of the mutants to grow, the genes identified are those that contribute to a gain or loss of fitness within the mutant pool grown in these conditions.

### Efflux and porin genes involved in ciprofloxacin susceptibility

**Figure 1.**
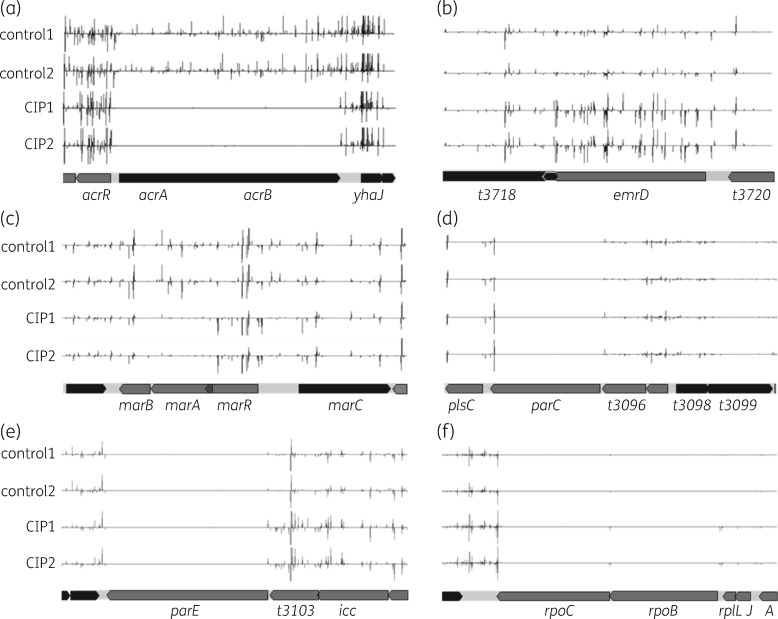
Mutant changes across some genetic locations following growth supplemented with ciprofloxacin. Distribution of nucleotide sequence reads generated by TraDIS at six different *S*. Typhi genetic loci following growth supplemented with and without ciprofloxacin, viewed using Artemis.^56^ At the bottom of each panel, genetic maps indicate the positions of the genes coded by the forward (dark grey) or reverse (light grey) DNA strands. Above each genetic map are four location and quantification plots, one for each duplicate experiment treated with ciprofloxacin (CIP1 and CIP2) or untreated (control1 and control2). Duplicates indicate the experimental reproducibility. Within the plots, fine vertical bars indicate the location of transposon insertion sites, with the length of the bar proportional to the number of nucleotide sequence reads (and therefore the relative number of mutants) that locate with that insertion site. Bars extending above the central axis indicate transposon insertion mutations oriented such that the kanamycin resistance determinant encoded by the transposon is transcribed in the left to right direction, and those below indicate the reverse orientation. (a) Transposon insertions within the *acrAB* genes coding for efflux are lost in the presence of ciprofloxacin, whilst mutants with insertions in the *acrR* gene, which codes for a repressor of *acrAB*, increase in numbers (log_2_FC = 1, i.e. by 2-fold). (b) Increased transposon mutants for the EmrD efflux system following growth in the presence of ciprofloxacin. (c) Increased insertion mutants in the *marA* repressor, *marR*, following growth with ciprofloxacin, oriented in the reverse direction such that transcriptional readthrough from the transposon will be into the *marA* gene; also reduced mutants in *marB*. (d) Insertions within the last codon of *parC* increase in the presence of ciprofloxacin, as indicated by an increase in the height of the bar extended above the central axis at this location. These insertions are oriented such that the transposon kanamycin resistance gene may reverse transcribe into the *parC* gene and thereby modulate expression through RNA interference. The bar extended below the central axis in this location is immediately outside of the *parC* gene. (e) Increased transposon insertions within the *t3103* gene with ciprofloxacin treatment may lead to altered expression of the *parE* gene coding for a subunit of topoisomerase IV, which is the secondary target of ciprofloxacin. (f) Transposon insertions into the last 25 bp of the *rpoC*, as well as immediately upstream of both *rpoB* and *rpoC*, increase with ciprofloxacin, suggesting that altered expression of DNA polymerase may play a role in susceptibility.

Those genes identified (Tables 1 and 2) that are already known to be involved in fluoroquinolone susceptibility^11^^,^^29–31^ serve to validate the TraDIS methodology and confirm that the fitness changes measured correlate with changes to ciprofloxacin susceptibility. For example, the genes coding for the AcrAB–TolC efflux transporter and the outer membrane porin OmpF were identified by our data as being important for *S*. Typhi susceptibility to ciprofloxacin (Tables 1 and 2; Figure 1a), as shown previously.^17^^,^^30^^,^^32–35^

Another efflux complex in *E. coli*, EmrAB–TolC, confers reduced susceptibility to several toxins,^36^ and this efflux complex is negatively regulated by EmrR. Our data indicate that in *S*. Typhi mutation of *emrR* conferred reduced susceptibility to ciprofloxacin (Table 2), as would be expected if *emrAB* became overexpressed due to loss of repression by EmrR. Mutations in *emrA* or *emrB* did not themselves increase susceptibility to ciprofloxacin, suggesting that *emrAB*-dependent efflux does not contribute significantly to loss of ciprofloxacin susceptibility in the presence of other efflux mechanisms. However, when overexpressed as a result of insertions into the *emrR* repressor gene, *emrAB*-dependent efflux does contribute to susceptibility, as has been observed also for *Salmonella enterica* serovar Typhimurium.^37^

In *E. coli*, *emrD* codes for MDR protein D, an efflux transporter with a wide range of structurally distinct substrates.^38^ Unexpectedly, in *S*. Typhi, mutations in *emrD* led to a gain in ciprofloxacin susceptibility, a result which was highly statistically significant (Table 2 and Figure 1b). This difference may be specific to *S*. Typhi, or it may reflect the coordinated regulation of multidrug efflux. It has been shown that disruption of efflux systems results in up-regulation of others in response,^39^ and it is possible that *emrD* knockout indirectly induces another ciprofloxacin exporter.

### Regulator genes involved in ciprofloxacin susceptibility

In *E. coli* and *S*. Typhimurium, the AcrAB–TolC efflux system and OmpF are regulated by the *marA*/*soxS*/*robA* regulon, and, in addition, by *ramA* in *S*. Typhimurium, which is absent from *E. coli.*^40^^,^^41^ MarA and Rob are positive regulators of the AcrAB–TolC efflux system, and MarA, in addition, is a negative regulator of the outer membrane porin, OmpF.^40^ Our data indicated that *marA* and *rob* insertion mutants of *S*. Typhi were more susceptible to ciprofloxacin (Table 1), as expected if the regulation by these genes in *S*. Typhi is similar to that for *E. coli* and *S*. Typhimurium.

Other regulators involved in expression of *acrAB* and *tolC* include *acrR* and *marR*, which were absent from our gene lists. In our data, *acrR* had a log_2_FC of 1.0 (average 1500 read counts in controls compared with 2800 for ciprofloxacin-treated cultures) and was therefore removed by our criterion of −1.5 ≥log_2_ FC ≥1.5, even though the *q* value for this gene was 7.4 × 10^−30^. Thus, the log_2_ FC limits employed to generate the gene lists of Tables 1 and 2 will miss some genes that may contribute to gain or loss of ciprofloxacin susceptibility, and *acrR* is one such gene (Figure 1), but will provide a more robust list of gene candidates.

For *marR*, there was little difference in the number of nucleotide sequence reads between the ciprofloxacin-treated and untreated growth conditions (log_2_ FC = 1.0). However, following growth with ciprofloxacin, there was an observable increase in the number of transposon insertions oriented such that transcription from the transposon kanamycin resistance gene could read through into *marAB* (Figure 1c). The Bio-Tradis software suite generates statistical data based on insertion sites on a gene-by-gene basis and, although the orientation of the insertions is known (from the DNA strand to which the nucleotide sequence reads locate), the software does not use this information. The enrichment of inserts in one orientation adjacent to genes supports the observation that TraDIS can be used to predict impacts of altered gene expression on phenotype.^42^

### Genes involved in DNA binding and repair

Like other fluoroquinolone antibiotics, ciprofloxacin acts by binding to DNA gyrase and topoisomerase IV, and results in DNA strand breaks.^43^^,^^44^ Hence, our whole-genome assay identified a number of genes coding for functions involved in DNA repair, such as *recN*, *recG*, *uvrD* and *xseA* (Table 1), which have been identified previously as contributing to diminished ciprofloxacin susceptibility in *E. coli*.^31^ In addition, we identified *hupA* and *hupB*, which encode the HU histone-like protein that binds to DNA recombination and repair intermediates, protecting them from exonuclease degradation.^45^ The HU protein is also known to control a regulon encompassing approximately 8% of the *E. coli* genome^46^ and may contribute to loss of ciprofloxacin susceptibility via several indirect regulatory mechanisms.

### Transposon insertions adjacent to genes coding for the fluoroquinolone target proteins and RNA polymerase genes

The ciprofloxacin targets, DNA gyrase and topoisomerase IV enzymes, act to maintain DNA topology in the bacterial genome, and their encoding genes (*gyrA* and *gyrB* for DNA gyrase, and *parC* and *parE* for topoisomerase IV) are essential.^21^^,^^42^^,^^47^ Consequently, transposon mutants of these genes are non-viable and are absent from our transposon mutant library, with the result that few or no sequence reads were identical to the nucleotide sequences of these genes. As a result, the *gyrA*, *gyrB* and *parE* genes were among the 12% of genes that were not assayed. However, there was a significant increase in mutants with insertions in the *parC* gene, indicating that these mutants displayed reduced susceptibility to ciprofloxacin. However, these insertion mutations were within the last 6 bp of *parC* and were oriented such that the transposon kanamycin resistance gene is reverse transcribed into *parC* (Figure 1d). Transposon insertions this close to the end of the *parC* gene probably do not result in inactivation, but may confer some loss in susceptibility to ciprofloxacin due to altered expression of *parC*, such as may occur through post-transcriptional gene silencing by RNA interference resulting from transcription of *parC* in the reverse direction.^48^

Gene *t3103* is known only as being conserved and hypothetical, but mutant numbers for this gene increased relatively in ciprofloxacin-treated cultures (Table 2). The *t3103* gene is upstream of the *parE* gene, and these insertion mutations may manifest their phenotype, at least in part, by modulating *parE* expression rather than being a direct result of insertion into the *t3103* gene (Figure 1e).

For the *rpoC* gene, encoding the essential RNA polymerase β′-subunit, as demonstrated by the absence of insertions across most of the gene (Figure 1f), the number of mutants increased relatively in the ciprofloxacin-treated cultures (Table 2). These insertion mutations were within the last 45 bp of the 3′ end of the *rpoC* gene (Figure 1f). Insertions in this part of the gene are unlikely to result in its inactivation, but probably manifest their phenotype through alteration of *rpoC* expression. There is also an increase in mutants with insertion 5′ to the *rpoB* gene (Figure 1f), indicating that these mutants, which probably alter transcription of the *rpoBC* gene, also display diminished ciprofloxacin susceptibility. RNA polymerase mutants have been shown previously to influence ciprofloxacin susceptibility by increasing MdtK-dependent efflux,^49^ and our data suggest that a subtle change in susceptibility may be achieved by modulation of RNA polymerase expression.

### Surface polysaccharide biosynthesis genes involved in ciprofloxacin susceptibility

Our experiments also identified at least 19 genes involved in polysaccharide and/or carbohydrate metabolism, in which transposon insertions reduced ciprofloxacin susceptibility (Table 2). Fifteen of these are involved in surface polysaccharide biosynthesis, including the *waa* (formerly *rfa*) genes *I*, *J*, *K* and *waaL*, coding for biosynthesis of the LPS outer core, and the LPS O-antigen biosynthetic genes *rfbE*, *I*, *K*, *M*, *S* and *rfbU*. Also included were the *galE* gene involved in UDP-galactose synthesis required both for LPS outer core and O-antigen. Other genes included those involved in the biosynthesis of enterobacterial common antigen (ECA), *rffM*, *rffD* and *wecB*,^50^ and *tviC* required for Vi-antigen biosynthesis.^51^ There are previous reports of LPS mutations resulting in changed susceptibility to fluoroquinolones, but our data provide a more comprehensive set of genes, and are the first to suggest a role for ECA and Vi-antigen.^17^^,^^30^^,^^34^^,^^52^

### Construction of mutants to validate candidate genes involved in diminished ciprofloxacin susceptibility

To confirm the validity of predictions from the TraDIS data, deletion mutations were constructed in the *S*. Typhi WT26 parent strain for the *hupA*, *tyrR*, *phoP*, *uvrD* and *xseA* genes, identified as candidates involved in ciprofloxacin susceptibility. All mutants showed a reduced MIC of ciprofloxacin, though the changes were small (Table 3). The mutants also showed a very small reduction in the MIC of the fluoroquinolone, ofloxacin, and the naphthyridone, nalidixic acid, which acts on GyrA in a similar way to the fluoroquinolones (Table 3). Both *uvrD* and *xseA* have been identified previously as being involved in ciprofloxacin susceptibility in *E. coli*.^31^

**Table 3.** MICs of ciprofloxacin (CIP), ofloxacin (OFX) and nalidixic acid (NAL) for defined mutants of *S.* Typhi

Strain	MIC (mg/L)		
	CIP	OFX	NAL
WT26	0.2	1.0	256
*ΔxseA*	0.1	0.85	128
*ΔphoP*	0.15	0.65	128
*ΔhupA*	0.15	0.65	128
*ΔtyrR*	0.15	0.85	256
*ΔuvrD*	0.15	0.65	128

For *ΔxseA*, *ΔphoP* and *ΔhupA* mutations, two mutants were generated independently and tested.

These results complement previous reports validating the TraDIS data and demonstrate their sensitivity for identifying loci involved in antibiotic susceptibility and other stressors. Whilst the phenotypic changes observed were small, these were for insertional inactivation mutations, and greater phenotypic changes may result from different types, or combinations, of mutation at these same loci.

### None of the pHCM1-encoded genes contributed to ciprofloxacin susceptibility

Many MDR strains of *S*. Typhi circulating in Southeast Asia harbour plasmid pHCM1 or similar derivatives.^25^^,^^26^ Thus, this plasmid was transferred into our *S*. Typhi Ty2-derived strain for TraDIS analysis experiments by conjugation. Plasmid pHCM1 is 218 kb and has 251 annotated genes, and our data indicate that none of these pHCM1-encoded genes contributes significantly to ciprofloxacin susceptibility.

### Concluding remarks

The continuing evolution of antibiotic resistance in clinically important human pathogens reduces treatment options for clinicians. A better understanding of the mechanisms of antibiotic resistance may provide opportunities for the identification of agents and implementation of practices to counter resistance, and it may also enable the prediction of when and where resistance will arise.^53^

By combining a very large transposon mutant library with a modified protocol for high-throughput nucleotide sequencing,^21^ we have screened about 88% of the genes in the genome of an *S*. Typhi Ty2 derivative for a role in the gain or loss of susceptibility to ciprofloxacin. However, most essential genes in which transposon insertions are not tolerated have not been assayed including, for example, *recA*, which is essential in *S*. Typhi but may also contribute to fluoroquinolone susceptibility.^21^^,^^54^ Whilst we have identified these genes by using insertional inactivation mutation, and, in the cases that we tested, MIC changes were small (Table 3), it is logical to consider that other types of mutations (for example, base or expression changes) in these same genes could also enhance or diminish the phenotype to a greater or lesser extent. Thus, using a whole-genome screen, we have found simultaneously many of the fluoroquinolone susceptibility mechanisms that have taken the previous decades to identify for *E. coli* and *Salmonella*, and new mechanisms, including those associated with genes of previously unknown function and phenotype, thus providing the first clues to their roles in bacterial biology.

## Supplementary Material

dkaa204_Supplementary_Data
