# Supplementary material for: A whole-genome screen identifies Salmonella enterica serovar Typhi genes involved in fluoroquinolone susceptibility
Source: J Antimicrob Chemother. 2020 Jun 8;75(9):2516–25. doi: 10.1093/jac/dkaa204 (PMC7443733; doi:10.1093/jac/dkaa204)
Supplement: dkaa204_Supplementary_Data [file dkaa204_supplementary_data.zip › Supplementary_data.docx]

**Supplementary data**

**Supplementary gene list**

An Excel file is supplied to supplement the gene lists provided by Tables 1 and 2 of the manuscript. This gene list was generated using less stringent filtering (-1 > log_2_FC > 1, q<0.001) than those employed to compile Tables 1 and 2, and so includes an additional 99 genes which may be of significance.

**Fastq files uploaded to European Nucleotide Archive**

Nucleotide sequence data fastq files generated using the TraDIS nucleotide sequencing protocol are available from the European Nucleotide Archive with study accession number PRJEB35758. Sample accession numbers as follows:

| Condition | Fastq filename | Accession number |
| --- | --- | --- |
| ctrl1 | ctrl1_3273-4354.fastq.gz | ERS4330755 |
| ctrl2 | ctrl2_3293-4334.fastq.gz | ERS4330756 |
| CIP1 | CIP1_3273-4334.fastq.gz | ERS4330757 |
| CIP2 | CIP2_3273-4334.fastq.gz | ERS4330758 |

BioTraDIS is available from: <https://github.com/sanger-pathogens/Bio-Tradis>

**Viewing transposon insertion site data using the *Artemis* software.**

The reference genome (an amalgamation of *S.* Typhi Ty2, Accession number AE014613, and plasmid pHCM1, Accession number NC_003384), may be viewed and compared with the plot files, generated by Bio-TraDIS and exemplified in Figure 1, using the *Artemis* software^1^ available from:

<http://sanger-pathogens.github.io/Artemis/Artemis/>

**Transposon mutant library and parent strain.**

The large transposon mutant library used for this work has been described previously and was constructed in an attenuated strain for safety reasons.^2-4^ The parental strain, WT26 pHCM1, has attenuating deletion mutations in *aroC*, *aroD* and *htrA*, and was constructed to include also a GyrA Ser83Phe substitution that confers reduced fluoroquinolone susceptibility.^5^ This is in common with many fluoroquinolone resistant isolates of *S*. Typhi and provides the first step to fluoroquinolone resistance.^6^. The parental strain also harbours the multiple antibiotic resistance plasmid pHCM1, itself and derivatives of which are common in MDR clinical isolates of *S*. Typhi circulating in south-east Asia.^7, 8^ Thus, the transposon mutant library was constructed in a strain which is safer but possesses characteristics like MDR clinical isolates allowing us, in theory, to investigate how other genes provide synergy with *gyrA* mutations in a comparable way to that which is occurring in clinical isolates.

**Mapping of sequence reads to identify transposon insertion sites in *S*. Typhi Ty2 and assessment of experimental variation.**

TraDIS generated over 16 million sequence reads from genomic DNA extracted from each culture (Tables S1). Over 13 million (80%) of the sequence reads were mapped to the *S*. Typhi Ty2 pHCM1 reference genome sequence (Tables S1). This allowed the identification of over 500,000 separate transposon insertion sites from each culture. On average this represents at least 1 insertion site every 10 bp in the 4.9Mbp *S*. Typhi Ty2 pHCM1 genome and over 100 independent inserts in an average size *S.* Typhi gene (Tables S1).

For the number of mapped sequence reads per gene, the correlation coefficient between the two control conditions (ctrl1 and ctrl2) is 0.9986, and that between the two ciprofloxacin supplemented conditions (CIP1 and CIP2) is 0.9997, confirming that there is very little experimental variation between biological replicate data sets.

**Number of genes assayed**

The Bio-Tradis software will not generate statistical data for genes that have too few mapped reads. Consequently, 4860 out of a total of 4895 (99%) annotated genes were analysed statistically. In order to remove from the data-set genes that may not have a sufficient number of mapped sequence reads, genes that had a log_2_ CPM (counts per million) value of less than 2.75 were removed. This takes out genes with fewer than 6.7 reads per million, and for our data-sets with over 13 million reads each, this therefore excludes genes with fewer than 90 mapped reads. This filter removed 535 genes, so that 4325 genes, or 88% of the genome, was effectively assayed by these experiments.

**Table S1. Summary of nucleotide sequence read parameters.**

| Culture^1^ | Total reads^2^ | 5-Tn (%)^3^ | Mapped reads (%)^4^ | Insertion sites^5^ |
| --- | --- | --- | --- | --- |
| ctrl1 | 16793620 | 14396994 (86) | 13517847 (94) | 517497 |
| ctrl2 | 22796340 | 20659694 (91) | 18708138 (91) | 624246 |
| CIP1 | 18823667 | 16985414 (90) | 15801486 (93) | 526321 |
| CIP2 | 18356833 | 16803298 (92) | 15722757 (94) | 521292 |

^1^LB broth cultures with added aro mix were grown supplemented with ciprofloxacin (CIP) at 0.05 mg/L or without (ctrl) in duplicate. ^2^Of the total number of nucleotide sequence reads generated, ^3^over 80% had the expected 10 bp of transposon sequence at the 5’-end, confirming these reads originated from a transposon insertion. Of these sequence reads, ^4^over 90% showed similarity to the sequence within the reference genome (*S.* Typhi Ty2, Accession number AE014613, combined with plasmid pHCM1, Accession number NC_003384), altogether ^5^confirming the locations of over half a million different insertions sites, which is, on average, one insertion site every 10 bp across the whole genome.

**Supplementary References**

1. Carver T, Harris SR, Berriman M et al. Artemis: an integrated platform for visualization and analysis of high-throughput sequence-based experimental data. *Bioinformatics (Oxford, England)* 2012; **28**: 464-9.

2. Langridge GC, Phan M-D, Turner DJ et al. Simultaneous assay of every Salmonella Typhi gene using one million transposon mutants. *Genome research* 2009; **19**: 2308-16.

3. Tacket CO, Sztein MB, Losonsky GA et al. Safety of live oral Salmonella typhi vaccine strains with deletions in htrA and aroC aroD and immune response in humans. *Infection and immunity* 1997.

4. Tacket CO, Sztein MB, Wasserman SS et al. Phase 2 clinical trial of attenuated Salmonella enterica serovar typhi oral live vector vaccine CVD 908-htrA in U.S. volunteers. *Infection and immunity* 2000; **68**: 1196-201.

5. Turner AK, Nair S, Wain J. The acquisition of full fluoroquinolone resistance in Salmonella Typhi by accumulation of point mutations in the topoisomerase targets. *J Antimicrob Chemother* 2006; **58**: 733-40.

6. Day MR, Doumith M, Do Nascimento V et al. Comparison of phenotypic and WGS-derived antimicrobial resistance profiles of Salmonella enterica serovars Typhi and Paratyphi. *J Antimicrob Chemother* 2018; **73**: 365-72.

7. Holt KE, Phan MD, Baker S et al. Emergence of a globally dominant IncHI1 plasmid type associated with multiple drug resistant typhoid. *PLoS Negl Trop Dis* 2011; **5**: 19.

8. Phan M, Kidgell C, Nair S et al. - Variation in Salmonella enterica serovar typhi IncHI1 plasmids during the global spread of resistant typhoid fever. *Antimicrob Agents Chemother* 2009; **53**: 716-27.
